# Short-Term Relapse Quantitation as a Fully Surrogate Endpoint for Long-Term Sustained Progression of Disability in RRMS Patients Treated with Natalizumab

**DOI:** 10.1155/2011/195831

**Published:** 2011-12-26

**Authors:** Y. C. Wang, A. Sandrock, J. R. Richert, L. Meyerson, X. Miao

**Affiliations:** ^1^Department of Biometrics, Biogen Idec, 14 Cambridge Center, Cambridge, MA 02142, USA; ^2^Neurology Clinical Development, Biogen Idec, 14 Cambridge Center, Cambridge, MA 02142, USA; ^3^Department of Biostatistics, Boston University School of Public Health, Boston, MA 02118, USA

## Abstract

Time to sustained worsening in the expanded disability status scale as the standard for evaluating the accumulation of disability has been used as a measure of clinical efficacy in many relapsing-remitting multiple sclerosis (RRMS) clinical trials. However, this measurement usually requires a large sample and long-term study to demonstrate the treatment effect. Annualized relapse rate or time to first relapse is also widely used as alternative measurements of clinical efficacy. A formal statistical validation of short-term relapse activity as a surrogate endpoint for long-term sustained progression of disability could potentially permit smaller, shorter, and less expensive clinical trials in RRMS. Four statistical validation/evaluation approaches consistently showed that relapse activity through one year of treatment serves as statistically valid surrogate endpoint for time to sustained progression of disability. The analysis demonstrates that long-term sustained progression of disability can be predicted by short-term relapse measures with 4 consistent validations of statistical approaches, including a formal statistical hypothesis test. This was demonstrated in a large phase III trial of natalizumab and showed that the beneficial clinical effect of natalizumab on sustained progression of disability at 2 years in patients with RRMS can be predicted by the total number of relapses at 1 year.

## 1. Introduction

Multiple sclerosis (MS) is an inflammatory disease of the brain and spinal cord characterized by focal areas of inflammation that lead to destruction of the myelin sheath and varying degrees of axonal injury. The typical inflammatory lesions of MS can occur throughout the central nervous system (CNS), but certain sites seem particularly vulnerable, such as the optic nerve, brainstem, spinal cord, and periventricular regions. In approximately 85% of people with MS the disease begins with relapses and remissions in disease activity. Recurrent attacks over decades of disease activity result in accumulating neurologic disability. In MS clinical trials, the standard for evaluating the accumulation of sustained disability, or the degree of neurologic impairment, is the EDSS, an overall disability status scale that has steps from 0 (normal) to 10 (death due to MS) [1]. It consists of 8 functional systems, each graded from 0 (normal) to 5 or 6 (maximum impairment), plus evaluation of gait. The limitations of the EDSS scale to assess disability are well known: it is heavily biased to locomotor function, it has poor sensitivity to change, especially at certain levels, and has only moderate inter- and intrarater reliability. For these reasons, this measurement usually requires a large sample size and long-term (2 to 3 years) study to demonstrate a beneficial treatment effect in clinical trials.

Other measures of disease activity include various MRI evaluations and measures of MS relapse activity. Because of the importance of this latter endpoint in clinical trials, multiple ways of quantifying relapse reductions have been utilized in previous studies, including ARR [2–7], proportion of relapse free patients [2–7], time to first relapse [2–4, 6, 7], severity of relapses [5–7], and mean number of new relapses during the study period [3, 4, 6, 7].

The large sample size and lengthy duration of study required to use disability progression as the primary outcome measure have made MS clinical trials operationally challenging. One goal of MS clinical trial research has been to identify more cost-effective ways of determining valid clinical responses to therapeutic interventions. Prior efforts to identify and validate surrogate markers for clinical disability outcomes in MS have been disappointing. Part of the difficulty has been the lack of a sufficient clinical database for evaluating these measures.

We have evaluated data from the large placebo-controlled AFFIRM trial of natalizumab [8] to explore the use of short-term relapse activity as a surrogate endpoint for longer-term sustained disability. We show that the clinical beneficial effect on sustained progression of disability at 2 years in patients with RRMS can be predicted by the one-year relapse rate, using validations of statistical approaches including a formal statistical hypothesis test.

## 2. Materials and Statistical Methods

### 2.1. Patients

Subjects with a diagnosis of RRMS as defined by McDonald et al. [9], and a baseline EDSS score between 0.0 and 5.0, inclusive, participated in a randomized, double-blind, placebo-controlled trial of natalizumab. Participants were between 18 and 50 years of age. Each had a cranial MRI scan demonstrating lesion(s) consistent with MS and had experienced at least one medically documented clinical relapse within the 12 months prior to randomization.

Of the 942 subjects enrolled in the study with 2 : 1 randomization, 315 were randomized to receive placebo with a mean followup of 1.79 years and 627 were randomized to receive natalizumab with a mean followup of 1.88 years. Apart from 3 subjects randomized to receive placebo who withdrew from the study prior to receiving randomized treatment, all subjects received at least 1 dose of study drug. Infusions of placebo or 300 mg natalizumab were scheduled to be administered every 4 weeks for up to 116 weeks. A total of 856 patients who completed the two-year study and who had no missing data regarding relapses at the 1-year timepoint were included in this analysis.

Baseline demography was balanced between the groups. The expanded disability status scale scores at baseline ranged from 0 to 6 (median of 2). Four subjects, two in each treatment group, had baseline EDSS scores greater than the upper limit of 5.0 specified in the entry criteria of the study.

### 2.2. Clinical Efficacy Outcome Measures

The primary clinical efficacy endpoint was the time to sustained progression of disability, as measured by at least a 1.0-point increase in the EDSS from baseline for patients entering with EDSS ≥ 1.0 or at least a 1.5 point increase for those with baseline EDSS = 0, each of which was sustained for 12 weeks. This endpoint was used to determine whether the treatment was effective in slowing the progression of disability. The coprimary endpoint in the study was ARR through the first year. Confirmatory analyses in the AFFIRM study, covering the entire 2-year study period included ARR, annualized relapse rate requiring steroid treatment, time to first relapse, and the proportion of patients who were relapse-free.

For the current analysis, we considered EDSS progression (noted as *T*) as the true clinical outcome and the total number of clinical relapses that a patient experienced through the first year as the candidate surrogate marker (noted as *S*) for EDSS progression.

### 2.3. Statistical Methodologies for the Validation of Surrogacy

We assessed the surrogacy of first-year relapses for the time to sustained progression of disability at 2 years by four well-recognized statistical approaches, including the statistical hypothesis-testing approach based on the *Prentice* criteria that has largely been used in surrogacy validation studies in MS [10–12] and that was recently applied to a study with similar aims in IFN-treated patients [13] and three quantification approaches for the effect of the surrogate endpoint.

#### 2.3.1. A Hypothesis Testing Approach Based on the Prentice Criteria

The Prentice criteria [14] are generally accepted as the statistical definition of a surrogate endpoint. A biomarker is considered a statistically valid surrogate endpoint if the following four conditions are satisfied: (1) the treatment has a significantly beneficial effect on the clinical endpoint, (2) the treatment has a significantly beneficial effect on the surrogate endpoint, (3) the clinical endpoint and surrogate endpoint are significantly correlated, and (4) the treatment effect on the clinical endpoint becomes statistically insignificant after adjusting for the surrogate endpoint.

In this paper, *T* denotes the true clinical endpoint, *S* denotes a potential surrogate endpoint, and *Z* denotes the treatment group. A biomarker *S* is considered a surrogate endpoint for a clinical endpoint *T* under the Prentice criteria if the triplet (*T*, *S*, *Z*) fulfills the following four conditions:

(1) $$\begin{array}{c} f\left(T\;|\;Z\right)\neq f\left(T\right), \end{array}$$

(2) $$\begin{array}{c} f\left(S\;|\;Z\right)\neq f\left(S\right), \end{array}$$

(3) $$\begin{array}{c} f\left(T\;|\;S\right)\neq f\left(T\right), \end{array}$$

(4) $$\begin{array}{c} f\left(T\;|\;S,Z\right)=f\left(T\;|\;S\right), \end{array}$$

where, for example, *f*(*T* | *Z*) is the conditional distribution of the true endpoint *T* given the treatment assignment *Z*, and *f*(*T*) is the unconditional distribution of the true endpoint *T*. When the condition (1) is satisfied, the treatment *Z* has a significant effect on the clinical endpoint *T*. Therefore, the first three criteria describe the dependence between any two of the triplet (*T*, *S*, *Z*) and they are preconditions for a biomarker to be considered as a candidate surrogate endpoint. The fourth criterion has been generally recognized as the statistical definition of surrogacy, which defines that the treatment effect on the clinical endpoint becomes statistically insignificant when it is adjusted by the surrogate endpoint, and we will refer to it as the Prentice criterion in the following sections.

In the natalizumab study, both the time to sustained disability at 2 years and ARR at 1 year were primary endpoints, and natalizumab was approved based on significant treatment effect on these two endpoints, hence the criterion (1) and (2) are satisfied. We will therefore focus on the verification of criteria (3) and (4). The validity of the third criterion was verified by modeling the time to sustained disability by the Cox proportional hazard model with total number of relapses at 1 year as the only predictor. The verification of the fourth criterion is based on modeling the time to sustained disability by the Cox proportional hazard regression model with treatment and total number of relapses at 1 year as covariates.

#### 2.3.2. Quantification Approaches

**Proportion of Treatment Effect Explained**

The proportion of treatment effect explained by surrogate endpoint (PTE) was proposed by Freedman et al. [15] to quantify how much the treatment effect on the clinical endpoint is captured by the surrogate endpoint based on an intuitive interpretation of the fourth Prentice criterion. PTE is computed as one minus the ratio of the regression coefficients of the treatment in a full model with both treatment and surrogate endpoints as the covariates, and a reduced model with only treatment as the covariate.

**Adjusted Likelihood Reduction Factor**

The adjusted likelihood reduction factor (LRF_a_) was proposed by Alonso et al. [16] to evaluate the treatment effect via surrogate endpoint based on the estimation of generalized correlation between the clinical endpoint and the surrogate endpoint. LRF_a_ is computed as

(5) $$\begin{array}{c} \text{LR}{\text{F}}_{\text{a}}=\frac{1-\exp\left\{-\left(\text{LRT}\left(Z+S:Z\right)\right)/n\right\}\;}{1-\exp\left\{-\left(\text{LRT}\left(Z+S:1\right)\right)/n\right\}}, \end{array}$$

where LRT(*Z* + *S* : *Z*) is the likelihood ratio statistic comparing the model with both *Z* and *S* as the covariates and the model with only *Z* as the covariate; LRT(*Z* + *S* : 1) is the likelihood ratio statistic comparing the model with both *Z* and *S* as the covariates and the model with only intercept.

**Proportion of Information Gain**

The proportion of information gained (PIG) by surrogate endpoint was proposed by Qu and Case [17] to quantify the effect of the surrogate endpoint via Kullback-Leibler information gain. The PIG is expressed as the ratio of information gained by the surrogate endpoint to the information gained by the surrogate endpoint and treatment together, as computed by

(6) $$\begin{array}{c} \text{PIG}=\frac{\text{LRT}\left(S:1\right)}{\text{LRT}\left(Z+S:1\right)}, \end{array}$$

where LRT(*S* : 1) is the likelihood ratio test statistic comparing the model with *S* as the covariate and the intercept model; LRT(*Z* + *S* : 1) is the likelihood ratio test statistic comparing the model with both *S* and *Z* as the covariates and the intercept model.

For the hypothesis testing approach, if the treatment effect becomes insignificant after adjusting for the candidate surrogate endpoint, then the candidate surrogate endpoint is considered a valid surrogate endpoint. For the three quantification approaches, a value close to 1 suggests good surrogacy.

## 3. Results

### 3.1. Satisfaction of the First and Second Prentice Criteria

In the clinical study report of natalizumab, the treatment effect on the time to sustained progression of disability at 2 years was statistically significant. The Kaplan-Meier estimate of percentage of subjects progressing by 2 years in the placebo group was 29% compared to 17% for the group that received 300 mg natalizumab. The comparison between the treatment group and placebo group was made using a Cox proportional hazards model adjusting for the baseline EDSS score and age (<40 versus ≥40). The hazard ratio obtained from this model was 0.58 (95% CI: 0.43, 0.77) indicating a 42% reduction in the risk of disability progression following treatment with natalizumab. The comparison between the treatment group and placebo group was highly statistically significant (*P* < 0.001; Table 1).

The coprimary endpoint ARR at 1 year was calculated using Poisson regression adjusting for the number of relapses in the previous year, baseline EDSS, the presence of Gd-enhancing lesions on T1-weighted MRI, and age. Subjects were censored at the time at which they added rescue treatment with an available MS treatment. ARR at 1-year for the placebo group was 0.805 (95% CI: 0.669, 0.969) compared to 0.261 (95% CI: 0.211, 0.323) for the group that received 300 mg natalizumab. This difference of 0.544 represents a 68% decrease in the ARR following treatment with 300 mg natalizumab versus placebo and was highly statistically significant (*P* < 0.001; Table 2). Therefore, both the first and second Prentice criteria are satisfied.

### 3.2. Satisfaction of the Third Prentice Criterion

To validate the satisfaction of the third Prentice criterion, we defined the optimal response with 0 relapse at 1 year and suboptimal response with at least one relapse at 1 year and tested whether the short-term relapse is a predictor for the long-term EDSS. The Wilcoxon rank sum test shows that the relapse at 1 year is a strong predictor for the EDSS at 2 years (*P* < 0.0001; Table 3). The Cox proportional hazard model also shows a strong association between ARR at 1 year and the EDSS at 2 years (*P* < 0.0001). The hazard ratio in Table 4 shows that a patient with one more relapse during the first year is 2.26-times more likely to develop sustained progression of disability compared to a patient with one less relapse during the first year. Thus, the third Prentice criterion is satisfied.

### 3.3. Assessing the Satisfaction of the Fourth Prentice Criterion

In the patient sample that entered the current analysis, based on the hypothesis testing approach, the Cox proportional hazard model shows that the treatment effect on EDSS at 2 years is statistically significant without adjusting for relapses (HR = 0.61, *P* = 0.0011) but is not statistically significant with adjusting for relapses (HR = 0.91, *P* = 0.5486), which suggests that treatment effect-related reduction in short-term relapse significantly delayed the long-term disability progression in RRMS patients. Thus the fourth Prentice criterion is also satisfied.

### 3.4. Conclusions

In practice, while compelling, the validity criterion is not easily checked for surrogacy. The Prentice criteria are much more easily verified than the validity criterion, yet are purported to imply the validity criterion. Our analysis shows that the four Prentice criteria are satisfied and the results suggest that the 1-year relapse rate is a statistically valid surrogate endpoint for progression of disability at 2 years. Furthermore, all three quantification approaches give a value close to 1, suggesting that the surrogacy is good and that the results are consistent with the Prentice criteria. Therefore, all four approaches support short-term relapse rate as a surrogate endpoint for long-term sustained progression of disability in RRMS patients. In other words, a majority of the treatment effect on long-term sustained progression of disability can be captured by short-term relapses, suggesting that relapses over the short term may be a reflection of the same pathophysiologic process that is leading to disability progression.

## 4. Discussion

Clinical trials of new immune modulators as disease modifying agents in MS have become larger and more expensive as the standards of care worldwide have included early and sustained treatment with existing therapies. Thus, control groups consisting of those receiving placebo alone have become both impractical and unethical in most clinical trial situations involving RRMS. Resultant clinical trial designs (e.g., add-on or head-to-head studies) have required larger numbers of subjects and/or longer observation periods, particularly when evaluating disability progression as an outcome measure. Thus, the identification of surrogate markers that may substitute for measures of disability progression has become an important goal in MS clinical trial research.

Most prior efforts to identify and validate surrogate markers for clinical disability outcomes in MS have been disappointing. Part of the difficulty has been the lack of a sufficient clinical database for evaluating these measures. The goal of the current study is to build a scientific link between long-term sustained progression of disability and short-term clinical relapses, which is operationally applicable in order to avoid large long-term studies when it is practical to do so.

We have evaluated data from the large placebo-controlled AFFIRM trial of natalizumab to explore the use of short-term relapse activity as a surrogate endpoint for long-term sustained disability. We show that the clinically beneficial effect on sustained progression of disability at 2 years in patients with RRMS is predicted by the one-year relapse rate using four consistent validations of the statistical approaches.

All four Prentice criteria were satisfied in this analysis. The criteria (1) and (2) were satisfied in the natalizumab study, as the treatment effects on both the time to sustained disability at 2 years and ARR at 1 year were statistically significant. The criterion (3) was satisfied, as both the Wilcoxon rank sum test and the Cox proportional hazard model showed strong associations between ARR at 1 year and the EDSS at 2 years. The criterion (4) was satisfied, as the Cox proportional hazard model showed that the treatment effect on EDSS at 2 years is statistically significant without adjusting for relapses but is not statistically significant when adjusting for relapses.

Furthermore, three quantification approaches supported the conclusion that a majority of the treatment effect on long-term sustained progression of disability can be captured by short-term relapses. The PTE, LRF_a_, and PIG evaluations all gave values close to 1, suggesting that the surrogacy is good and that the results are consistent with the Prentice criteria. Therefore, all four approaches support measurement of short-term relapses as a surrogate endpoint for long-term sustained progression of disability in RRMS patients.

It is possible that the responsiveness of this measure is specific for only some, but not all, treatment modalities. Natalizumab, fingolimod, and some of the interferons have demonstrated efficacy using outcome measures for both relapse rate and disability progression. However, glatiramer acetate has shown efficacy in reducing relapse rate but failed to demonstrate slowing of disability progression over a two-year period. Recently Sormani et al. [18] evaluated the link between responsiveness of relapse rate or MRI characteristics and responsiveness of disability progression using a pooled database consisting of data from 19 clinical trials of various therapeutic agents in RRMS. Eleven of these trials involved interferon beta, two involved natalizumab, and one trial each involved mitoxantrone, IVIg, alemtuzumab, cladribine, and azathioprine. Only two involved glatiramer acetate. It is quite likely that, because the data involving glatiramer acetate were derived from too small a percentage of the total to be reflected in the final outcome, those data were overwhelmed by data from trials involving agents that beneficially affected both relapse rate and disability progression. In any case, it is possible that the methodology described in the current report and in the report by Sormani et al. [18] will not be applicable to the study of all proposed disease modifying agents.

In conclusion, the data reported here, together with the published study of Sormani et al., strongly suggest that in studies of therapeutic agents that favorably affect both relapse rate and disability progression in RRMS, measurement of relapse rate serves as a reliable surrogate marker for disability progression. Data from the pivotal trial of glatiramer acetate in RRMS indicate that this relationship may not be evident for all therapeutic modalities, however. This suggests that, while disability progression should continue to be directly measured in pivotal phase III trials in RRMS, once a therapy has been shown to benefit both relapse rate and disability progression, subsequent phase 4 trials may be able to utilize measures of relapse rate as a reliable surrogate for disability progression.

## Figures and Tables

**Table 1:** Summary of time to sustained progression of disability at two years as measured by increase in EDSS—ITT population.

	Placebo	Natalizumab	*P* value^a^
Number of subjects randomized	315 (100)	627 (100)	
Number of subjects progressed	84 (27)	104 (17)	
Time (yr) to progression^b^			
Mean and 95% CI	1.78 (1.72, 1.84)	1.90 (1.87, 1.94)	
Estimated proportion of progression at 2 years^b^	0.29	0.17	
Hazard ratio (natalizumab/placebo) and 95% CI	0.58 (0.43, 0.77)		<0.001

Note: sustained progression of disability is defined as at least a 1.0-point increase on the EDSS from a baseline EDSS ≥ 1.0 sustained for 12 weeks or at least a 1.5-point increase on the EDSS from a baseline EDSS of 0 sustained for 12 weeks.

^a^: *P* value assessing the difference between the treatment groups, from Cox proportional hazards model, adjusted for baseline EDSS values and age (<40 versus ≥40).

^b^: Estimated time to progression and proportion of subjects with progression based on the Kaplan-Meier product limit method.

**Table 2:** Annualized relapse rate—ITT population—one-year analysis.

	Placebo	Natalizumab	Rate Ratio (95% CI) and *P* value (a)
Number of subjects randomized	315 (100)	627 (100)	
Number of subjects with a relapse	135 (43)	132 (21)	
Number of relapses^b^			
0	180 (57)	495 (79)	
1	76 (24)	111 (18)	
2	42 (13)	17 (3)	
3	8 (3)	4 (<1)	
≥4	9 (3)	0	
Total number of relapses	224	157	
Total subject-years followed	320.1	652.6	
Unadjusted annualized relapse rate^c^	0.700	0.241	
Adjusted annualized relapse rate	0.805	0.261	0.325
(95% CI)^a^	(0.669, 0.969)	(0.211, 0.323)	(0.256, 0.412)
			<0.001
Subject relapse rate^d^			
Mean	0.778	0.262	
Median	0.000	0.000	

Note: the analysis includes relapses and time on the study up to the time that alternative MS medication is added.

^a^: From Poisson regression, adjusted for the number of relapses in the one year prior to study entry, baseline EDSS (≤3.5 versus >3.5), presence of Gd lesions (present versus absent), and age (<40 versus ≥40).

^b^: Numbers in parentheses are percentages.

^c^: The total number of relapses that occurred during the study divided by the total number of subject-years followed in the study.

^d^: The number of relapses for each subject divided by the number of years followed in the study for that subject. Mean and median across all subjects are presented.

**Table 3:** EDSS score at 2 years in patients with optimal (0 relapse) or suboptimal responses (≥1 relapses) at 1 year—ITT population.

	Optimal	Suboptimal	*P* value^a^
EDSS score			
*n *	637	218	
Mean (SD)	2.093 (1.283)	3.016 (1.605)	
Median	2.0	3.0	<0.0001
Range	0, 6.5	0, 8.0	

^a^: *P* value is from Wilcoxon rank sum test.

**Table 4:** Validation of 1-year relapses as the surrogate endpoint for EDSS at 2 years.

		Hypothesis testing approach*								
		Association between relapse and EDSS		Treatment effect on EDSS w/o adjusting relapse		Treatment effect on EDSS with adjusting relapse		Quantification approaches		
True endpoint	Surrogate endpoint	HR	*P* value	HR	*P* value	HR	*P* value	PTE	LRF_a_	PIG
EDSS at 2-yr	Relapse at 1-yr	2.26	<0.0001	0.61	0.0011	0.91	0.5486	0.801	0.892	0.995

*: All Cox proportional hazards model for the time to sustained disability are adjusted for the baseline EDSS score and age group (<40 versus ≥40).

*Analysis was restricted to the patients who had no missing values of the endpoints.
